# Alternative splicing and differential gene expression in colon cancer detected by a whole genome exon array

**DOI:** 10.1186/1471-2164-7-325

**Published:** 2006-12-27

**Authors:** Paul J Gardina, Tyson A Clark, Brian Shimada, Michelle K Staples, Qing Yang, James Veitch, Anthony Schweitzer, Tarif Awad, Charles Sugnet, Suzanne Dee, Christopher Davies, Alan Williams, Yaron Turpaz

**Affiliations:** 1 Affymetrix, Inc., 3420 Central Expressway, Santa Clara, CA 95051, USA

## Abstract

**Background:**

Alternative splicing is a mechanism for increasing protein diversity by excluding or including exons during post-transcriptional processing. Alternatively spliced proteins are particularly relevant in oncology since they may contribute to the etiology of cancer, provide selective drug targets, or serve as a marker set for cancer diagnosis. While conventional identification of splice variants generally targets individual genes, we present here a new exon-centric array (GeneChip Human Exon 1.0 ST) that allows genome-wide identification of differential splice variation, and concurrently provides a flexible and inclusive analysis of gene expression.

**Results:**

We analyzed 20 paired tumor-normal colon cancer samples using a microarray designed to detect over one million putative exons that can be virtually assembled into potential gene-level transcripts according to various levels of prior supporting evidence. Analysis of high confidence (empirically supported) transcripts identified 160 differentially expressed genes, with 42 genes occupying a network impacting cell proliferation and another twenty nine genes with unknown functions. A more speculative analysis, including transcripts based solely on computational prediction, produced another 160 differentially expressed genes, three-fourths of which have no previous annotation. We also present a comparison of gene signal estimations from the Exon 1.0 ST and the U133 Plus 2.0 arrays.

Novel splicing events were predicted by experimental algorithms that compare the relative contribution of each exon to the cognate transcript intensity in each tissue. The resulting candidate splice variants were validated with RT-PCR. We found nine genes that were differentially spliced between colon tumors and normal colon tissues, several of which have not been previously implicated in cancer. Top scoring candidates from our analysis were also found to substantially overlap with EST-based bioinformatic predictions of alternative splicing in cancer.

**Conclusion:**

Differential expression of high confidence transcripts correlated extremely well with known cancer genes and pathways, suggesting that the more speculative transcripts, largely based solely on computational prediction and mostly with no previous annotation, might be novel targets in colon cancer. Five of the identified splicing events affect mediators of cytoskeletal organization (ACTN1, VCL, CALD1, CTTN, TPM1), two affect extracellular matrix proteins (FN1, COL6A3) and another participates in integrin signaling (SLC3A2). Altogether they form a pattern of colon-cancer specific alterations that may particularly impact cell motility.

## Background

Alternative splicing of mRNA transcripts is one mechanism by which genomic complexity is generated from the surprisingly low number of genes currently estimated from the human genome sequence. The fraction of human genes subject to alternative splicing has risen from 5% in early predictions to at least 75% in a recent genome-wide exon study (Clark *et al*., in prep.). There are examples of hundreds of alternative splicing events from a single gene, which may affect function by adding or deleting functional domains, changing affinities, and altering mRNA stability. Variable transcripts from a single gene are produced combinatorially through the selection of cassette exons, mutually exclusive exons, retained introns, alternative 3' or 5' splice sites, and alternative promoters or polyA sites [1].

Specific alterations in splicing patterns have been found in association with cancers, many of which may play a functional role in transformation, motility and metastasis of tumor tissue. Alternative splicing appears to affect key aspects of neoplasms by altering hormonal signaling, apoptosis and mediators of cell-cell and cell-matrix interactions. Modifications in functionality may be generated, for example, through the deletion of a signaling domain, increased affinity for messenger ligands, or change in affinity or activity toward extracellular components. The latter frequently results in increased cell migration and invasion (For general reviews, see [2,3]). Although in most cases the relationship between specific splicing events and the etiology of cancer is largely unproven, alternative splicing presents novel targets for diagnostic and therapeutic measures.

Abnormal splicing of several genes has been observed in tumors of colorectal origin, including *CD44 *[4,5], *MUC2 *[6], *SRF *[7], *NCAM *[8], *MLH*, *MSH *[9] and members of the Wnt pathway [10]. Some of these alternately spliced gene products may have therapeutic utility as markers of the progression of the disease or as drug targets; however there are insufficient data supporting their relevance in larger clinical populations. Individual patient differences, tissue complexity and lack of tools for comprehensive analysis of splice variation have made the task challenging.

We have analyzed both differential gene expression and alternative splicing in a small collection of colon cancers employing a microarray (GeneChip Human Exon 1.0 ST) targeting over a million putative exons. The exons can be virtually reassembled into over 250,000 transcripts according to a range of annotation sources. The inclusiveness of the array design allowed a flexible gene expression analysis, initially targeting high confidence transcripts and then extending to a more speculative set.

Cancer-specific splice variations were detected with experimental algorithms and candidate events were subsequently validated with RT-PCR. We found several splice variants, some of which have been previously associated with cancer, and several which have not. The majority of the identified splicing events affect mediators of cytoskeletal organization, the extracellular matrix or integrin signaling, and may be involved in cell migration and invasion.

## Results

### Array Design

The GeneChip Human Exon 1.0 ST array was designed to be as inclusive as possible at the exon level, deriving from annotations ranging from empirically determined, highly curated mRNA sequences to *ab-initio *computational predictions [11]. The array contains approximately 5.4 million 5-μm features (probes) grouped into 1.4 million probesets interrogating over one million exon clusters (exon annotations from various sources that overlap by genomic location). By comparison, the Affymetrix U133 Plus 2.0 gene expression array contains 1.3 million 11-μm features comprising 54,000 probesets. A Probe Selection Region (PSR) represents a region of the genome (assembly HG16, Build 34) predicted to act as an integral, coherent unit of transcriptional behavior. In many cases, each PSR is an exon; in other cases, due to potentially overlapping exon structures, several PSRs may form contiguous, non-overlapping subsets of a true biological exon. The median size of PSRs is 123 bp with a minimum of 25 bp. Probes were not included for some PSRs because the PSR might be too small or contain highly repeated or otherwise problematic sequence. About 90% of the PSRs are represented by 4 probes (a "probeset"). Such redundancy allows robust statistical algorithms to be used in estimating presence of signal, relative expression, and existence of alternative splicing.

The Exon Array does not include a paired mismatch feature for each perfect match feature, rather surrogate mismatch intensities are derived from 1000 pooled "antigenomic" probes with the same GC content as each perfect match feature [11]. Antigenomic sequences are not present in the human, rat or mouse genomes.

The Exon Array also serves as a gene-level expression array. The median number of probes for each RefSeq gene is 30–40 distributed along the entire length of the transcript, as compared to 11 probes mostly at the 3' end in the GC U133 Plus Array.

The plethora of exon architectures (e.g., cassette exons, mutually exclusive exons, alternative splice sites, alternative transcriptional starts and stops), the variations in confidences of transcript annotations, and the necessity of rapidly incorporating new genomic knowledge has led to a dynamic design for reconstituting exons into genes. A set of rules was created for virtually assembling the probesets (exon-level) into transcript clusters (gene-level) based on the confidence level of the supporting evidence and the juxtapositions of the exon borders [11]. In general, exons were merged into clusters depending on whether and how many higher confidence clusters or exons they overlap, whether they have common exact splice sites, and whether single orphan exons are bounded within another annotation.

The mapping between probesets and transcript clusters is defined by meta-probeset lists (in order of decreasing confidence):

A) "Core" (17,800 transcript clusters): RefSeq and full-length GenBank mRNAs;

B) "Extended" (129K transcript clusters): cDNA transcripts, syntenic rat and mouse mRNA, and Ensembl, microRNA, Mitomap, Vegagene and VegaPseudogene annotations;

C) "Full" (262K transcript clusters): *ab-initio *predictions from Geneid, Genscan, GENSCAN Suboptimal, Exoniphy, RNAgene, SgpGene and TWINSCAN

The combination of merging rules and metaprobe sets means that some transcript clusters in the Extended and Full sets may contain regions (probesets) that derive from annotations of different confidence levels. Of the 1.4 million probesets, approximately 290,000 are supported by full-length mRNAs (i.e., the Core set).

### Gene-level signal comparison between the U133 Plus 2.0 and Exon arrays for normal tissue

Prior to analyzing the colon cancer data, we present a brief comparison of the gene level signals from the exon array and Affymetrix's standard gene expression array, the U133 Plus 2. For this, we utilize a subset of data from a panel of 11 normal tissues that was assayed in parallel on both arrays. The approach was to compare the relative signals from differentially expressed probesets that target the same transcript clusters on both arrays.

Significant genes were determined by pair-wise comparisons between each set of the 11 tissues with p-values at 0.05, 0.001 and 10^-8^. Genes were accepted into the analysis if they showed significant changes in at least one tissue type on both arrays and could be mapped to the same transcript cluster. At a p-value of 0.05, 7829 probesets from the U133 Plus 2 array and 5507 transcript clusters from the Core meta-probeset of the exon array were accepted and mapped to each other. On average, approximately 1.4 probesets from the U133 Plus 2 array mapped to each transcript cluster on the exon array. This disparity in numbers results because the U133 Plus 2 array frequently has multiple independent probesets targeting the same transcript cluster, whereas the probesets are clustered and summarized into a single gene signal estimate on the exon array. For the 1654 cases of multiple U133 Plus 2 probesets interrogating the same gene, the mean of the individual signals was used as the signal estimate.

**Figure 1:**
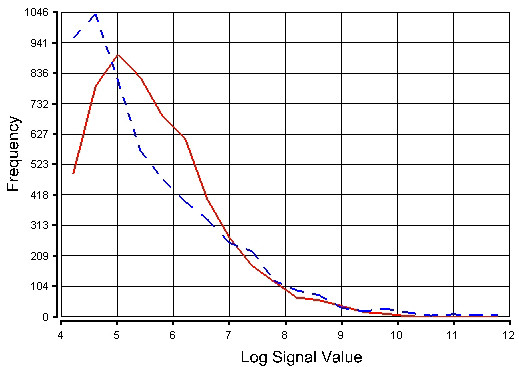
**Frequencies of signal values from the Exon and U133 Plus 2 Arrays**. The distribution of log_2 _(signal + 16) values of transcript clusters are shown for the Exon Array (solid line) and the U133 Plus 2 Array (stippled line) for breast tissues.

The distribution of signals for these 5507 transcript clusters from the two array types for the breast tissue samples are shown in Fig. 1. To reduce the possible confounding effects of alternative splicing on the signal estimation, breast tissue was chosen rather than, e.g., brain or testes, which typically show high levels of splice variation. The most striking indication from the figure is the apparent shift of low expression signals from U133 Plus 2 (a logged signal of less than 5) to a relatively higher expression level (logged signals between 5 and 7) in the exon array. The U133 Plus 2 also seems to give marginally higher signal at high expression levels.

**Figure 2:**
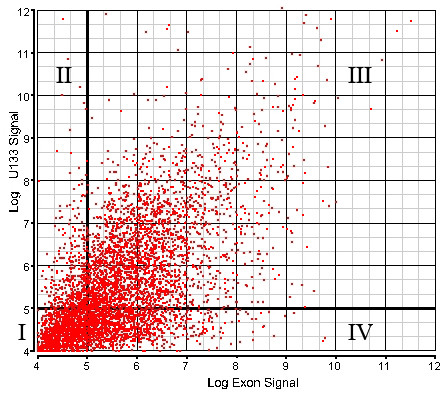
**Pairwise comparison of signals from the Exon and U133 Plus 2 Arrays**. Each dot represents the log_2 _(signal + 16) values for the same transcript cluster from the Exon Array (x-axis) and the U133 Plus 2 Array (y-axis) for breast tissues. Bold lines at signal value 5 divide the plot into quadrants I-IV for reference.

Fig. 2 shows the pairwise signal pattern between transcript clusters from the two arrays. For convenience, the plot has been divided into quadrants at a logged signal of 5 (about double the background signal). The apparent shift of low expression signals in U133 Plus 2 to higher expression in the exon array is confirmed by the scatterplot. Quadrant IV (low signal on U133 Plus 2 and higher signal on the exon array) contains 1158 transcript clusters while the converse situation in Quadrant II (low signal in the exon array and higher signal on U133 Plus 2) contains only 448 transcript clusters. The overall correlation of the signals is 0.60, and the linearity of the data appears to be skewed by the shift of the signals at the low end. As the p-value for gene selection becomes more stringent, the correlation increases (data not shown).

### Gene-level expression changes in colon cancer for the Core and Full gene sets

The initial analysis of expression changes in colon cancer targeted Core genes, composed of nearly 17,800 transcript clusters based on highly confident supporting evidence. Subsequent to ANOVA analysis, a threshold cutoff was set to p-values less than 0.0015 and at least a 2-fold geometric change in gene-level expression between normal and tumor samples. This yielded 134 significantly up-regulated and 26 down-regulated genes in the tumor samples (see Additional file 1).

The most heavily represented classes of over-expressed genes in the tumor samples encompass mitosis, the cell cycle, apoptosis, cell proliferation, and DNA repair and recombination (34 altogether). One large group involves RNA processing (16 genes), including at least 4 participating in RNA splicing. Components or modulators of the extracellular matrix are also heavily represented (14 genes), many of which have been implicated in cell invasion and metastasis.

Many of the significantly down-regulated genes represent normal colon functions that are lost as the tissue becomes undifferentiated, such as Aquaporin 8, which is normally present only in colon and pancreas [12]. However, the decreased expression of two hormones, Somatostatin and PYY, and HIF3A, a negative regulator of hypoxia-inducible genes, may actively potentiate carcinogenesis [13-15].

**Figure 3:**
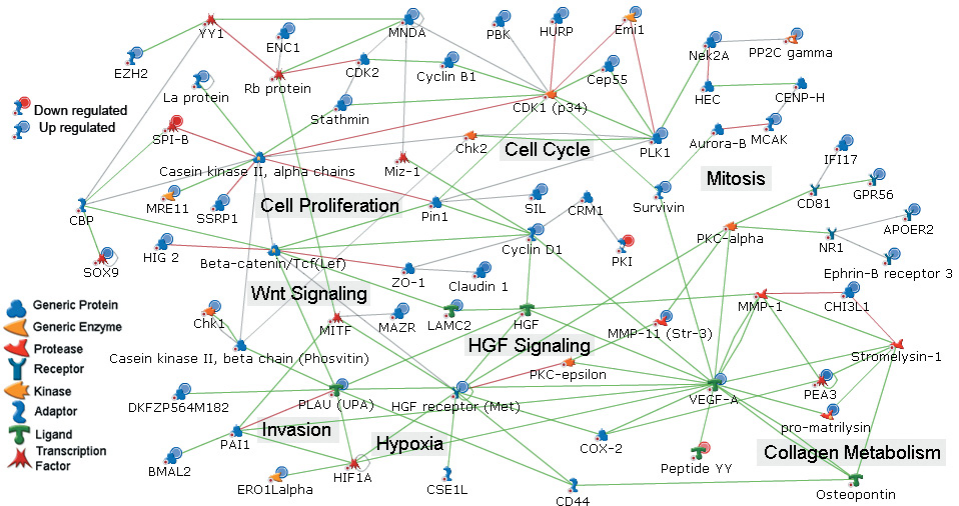
**A dense network of molecular interactions containing differentially expressed Core genes in colon cancer**. Over- or under-expressed genes, respectively, are indicated by a blue or red disk above-right of the gene-product icon. Green or red lines represent stimulatory or inhibitory interactions, respectively. Many transcription factors (e.g., p53) and signaling kinases (e.g., ERK1) are excluded due to their multiplicity of connections. The general partitioning of the nodes into canonical pathways are shown as bold text.

Pathway analysis (Fig. 3) indicates that the over-expressed genes largely populate a dense network involved in cell proliferation and migration, including VEGF-A, PLAU and MET (HGF receptor), which have been implicated in control of cellular invasion and in colon cancer specifically [16,17]. In this mapping, VEGF-A lies at the center of a network involved in collagen metabolism. Control of cell proliferation is centered around casein kinase IIa and β-catenin, which is a key mediator of Wnt signaling [18]. Other functions represented in the network include anti-apoptosis, proteolysis, chemotaxis and response to hypoxia.

The gene-level analysis was repeated with the more speculative Full metaprobe set representing 262,000 transcript clusters, i.e., putative genes generated from exon clusters without strong empirical support. ANOVA analysis produced 290 genes with differential expression between normal and cancerous tissue. Excluding 130 genes that were also found in the Core gene analysis leaves 160 genes found only in the expanded analysis, of which 39 have some annotation (Additional file 2). Of the 121 remaining completely unannotated putative genes, 38 have significant expression (a threshold PLIER signal of 15 in 50% of the samples) in at least one tissue type.

### Workflow for the detection of splice variants

**Figure 4:**
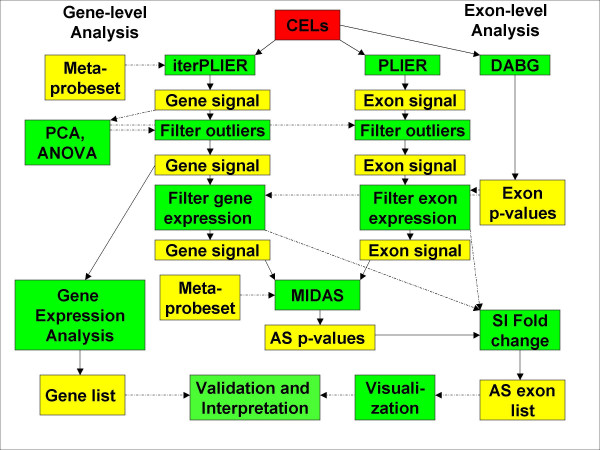
**Workflow for parallel gene-level and exon-level analyses**. The workflow illustrates two parallel lines of analysis corresponding to gene-level (left) and exon-level (right) signal processing. The analyses converge at the level of MIDAS or the Splicing Index, which implement statistical testing for alternative splicing. Signal estimation and MIDAS occur within the ExACT software and the filtering is accomplished mainly with simple Perl scripts. Yellow boxes represent files or data sets and green boxes represent processes or programs. Solid lines represent the main data flow and dotted lines are auxiliary flows, mainly for filtering.

We introduce here a workflow (Fig. 4) for detecting alternative splicing events between normal and cancerous samples using algorithms that normalize exon signals to the gene-level signal for each sample, followed by a tissue-specific ANOVA (in MIDAS) or t-test (in the Splicing Index [SI])[19,20]. Given the limited number of samples, the various stages of cancer progression and the aberrant behavior of several sample pairs, the data were expected to be noisy. To compensate, the data were filtered by multiple methods at both exon-level and gene-level to reduce problematic cases. Each combination of filtering and algorithms produced some viable candidates, but the most stringent filtering generally showed the best consistency with PCR validation results. The method ultimately producing the highest proportion of true positives was to retain only: a) exons with a DABG p-value < 0.05, b) genes with a signal > 70, c) exons with a log ratio between tissues (i.e., the gene-level normalized fold change) > 0.5, d) Splicing Index p-values < 0.005 and e) Core exons.

**Figure 5:**
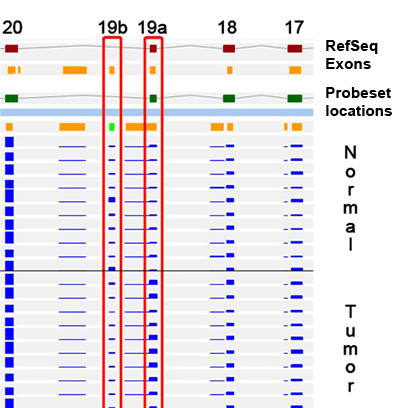
An example of probeset-level intensities from two mutually exclusive exons (19a and 19b) of *ACTN1 *in a genomic context. The probeset signals are normalized for each sample to the median signal for that sample across all the probesets in the view. This candidate splicing event was confirmed by RT-PCR. (BLIS viewer from Biotique.)

This filtering method generated 189 putative splicing events (Additional file 3), including 7 of the 9 genes eventually unambiguously validated by PCR (see below). Another confirmed splicing event in *CTTN *barely missed the threshold with a fold change of 0.47. Most of the positively validated splicing events were concentrated high in the p-value list, with *COL6A3 *and *ACTN1 *as the top two. The clearest PCR validations generally resulted from candidates (e.g., *ACTN1*, *CALD1*) that consistently ranked highly regardless of the algorithm or filtering method used. Conversely, most of the negatively performing genes in PCR validation resulted from attempts to extend the limits of our search or to test other filtering approaches. In each case, the candidates were evaluated and further filtered by visualizing the probeset intensities within the genomic context (Fig. 5).

### RT-PCR validation of differential splicing in colon cancer

**Figure 6:**
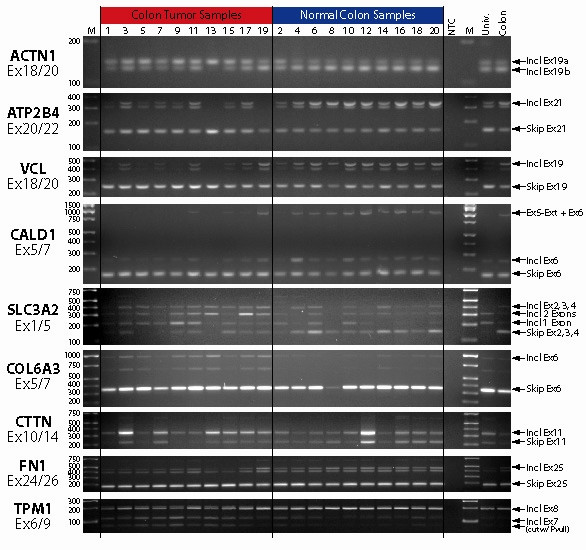
**PCR validation of candidate splicing events**. PCR products are derived from forward/reverse primers in well-annotated flanking regions as indicated under the gene name. The interpretation of each change in mobility is given to the right ("Incl", include; "Ext", extended). The sample numbers match those in the sample data information (see Methods). Note that in several cases, an unlabeled doublet band results from a heteroduplex forming between the PCR products. Controls are "Universal Total RNA" (Clontech), an independent colon RNA sample (BioChain) and a No Template Control ("NTC"). "M" is a DNA ladder marker.

In order to empirically assess the presence or absence of putatively spliced exons, PCR primers were designed on adjacent constitutively expressed exons, in some cases spanning several target exons. Some of the validated differential splicing events are shown in Fig. 6. One of the two tumor samples characterized as poorly differentiated was consistently prototypical of the alternative splicing patterns of these genes (compare the tumor-normal pair in Lanes 13 and 14). A common artifact affecting RT-PCR of alternatively spliced mRNAs appears in some of the gels (*ATP2B4*, *VCL*, *FN1*) as an unidentified band migrating just below the largest band. This band represents a heteroduplex of the two alternatively spliced forms [21,22].

Altogether, forty-three genes were tested by RT-PCR, drawn primarily from the filtering method described above. Nine candidate genes (Table 1) showed clear differential alternative splicing in colon cancer relative to normal colon tissue, with two events occurring in *COL6A3*. An additional five genes showed positive results for discriminatory splicing but with some ambiguity. Of the other tested genes, 13 exhibited alternative splicing but were not distinctive between normal and cancerous tissues, and 16 showed no evidence of alternative splicing (Additional file 4). In total, approximately one-third of the 43 tested splicing events gave positive results in the PCR validation. Each of the validated splicing events derive from the Core annotation set, and are found in RefSeq or GenBank, usually as cassette-type or mutually exclusive exons.

Table 1 also includes the gene-level fold change for each of the validated splicing events since strong tissue-specific differences in gene expression may cause misleading results in the RT-PCR validations. However, all of the strongly validated events demonstrated a fold change of less than 2 (two of the ambiguous events were > 2), and none of these genes appeared as significantly differentially expressed in the gene-level analysis.

### Validated splicing events

Alpha-actinin 1 (*ACTN1*) and vinculin (*VCL*) encode cytoskeletal elements that interact with actin and probably participate in the attachment of the cytoskeleton to the membrane. Exons 19a and 19b of *ACTN1 *appear to be mutually exclusive exons, with 19a predominant in tumor samples and 19b predominant in normal samples (Fig. 6). Exon 19 of *VCL *is a cassette exon that tends to be skipped in these tumor samples.

ATP2B4 belongs to the family of ion transporters that exhibits many isoforms with alternatively spliced exons regulated in a tissue-specific manner. Exon 21 is a known cassette-type exon that is found here in normal colon tissue but is relatively reduced in tumor samples. ATP2B4 plays a role in cellular calcium homeostasis that may be altered in tumor cells in parallel to the loss of ion transporters seen at the gene level.

Variants of caldesmon (CALD1), a structural element linking myosin and actin filaments, tend to include exon 6 and an extended exon 5 in normal cells. *SLC3A2 *encodes the heavy chain of a heterodimeric solute carrier, but also participates in integrin signaling [23,24]. Transcripts for *SLC3A2 *are shifted to a higher molecular weight, indicating the inclusion of various combinations of exons 2, 3 and 4 in tumor samples that are much less prevalent in normal tissue. Transcripts for the matrix protein Collagen IV-3A (COL6A3) tend to include the cassette exons 3, 4 (not shown) and 6 (Fig. 6) in tumor samples to a greater extent than in normal samples.

Cortactin (CTTN) also has roles both in cell adhesion and organization of the cytoskeleton. Exon 11 is a cassette exon that appears to be equally likely to be included or skipped in normal tissue, but in the tumor samples, the predominant form is inclusion of Exon 11. Fibronectin (FN1) can be found in the extracellular matrix or at the cell surface, and has a role in cell adhesion and migration. A cassette exon, Exon 25, is more prevalent in normal tissues than in tumor samples.

Tropomyosin 1 (TPM1) is also an actin-binding protein, has a number of known splice variants, and is involved in the contraction of muscle and in the cytoskeleton of non-muscle cells. Since Exons 7 and 8 of *TPM1 *are mutually exclusive exons of approximately the same size, the alternative amplificates were distinguished by a restriction enzyme that cuts Ex7, but not Ex8. The fragments of Ex7 appear as two faster moving bands that are more prevalent in the tumor samples.

Densitometric scans of the gel bands were used to quantitate the observed splice variants (Additional file 5). The ratio of the intensity of the alternative bands was calculated and compared across the tissue types as a log ratio. The densitometric analysis suggests that the strongest and most consistent changes occurred in *ACTN1*, *ATP2B4*, *VCL *and *CALD1*. *TPM1 *and *CTTN *also showed strong splicing differences between the tissues, but with higher variability. All of the tested events show a p-value of < 0.05 in the t-test across the tissues except for Ex6 of *COL6A3*, for which the splice variants are more apparent visually than in the scan.

### Validation of previously reported splicing events

**Figure 7:**
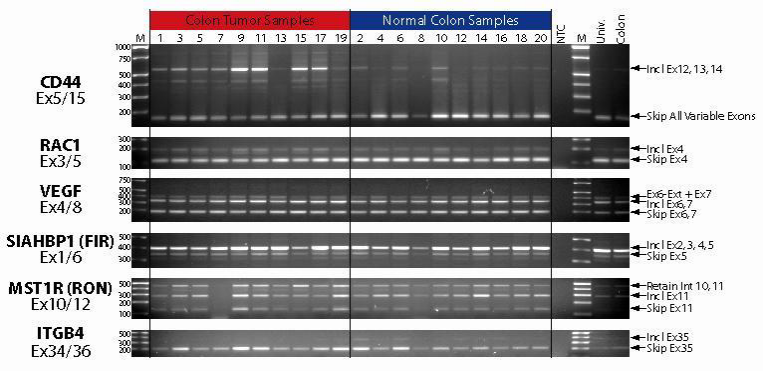
**PCR validation of previously reported colon cancer-specific splicing events**. See Fig. 6 for description.

We also tested for the presence of several alternative splicing events that were previously reported in colon cancers [25-29], but were not strongly indicated by our statistical analysis. The expected splicing events in *Rac1*, *VEGF*, *SIAHBP1*(*PUF60*) and *MST1R *were weak or absent in these samples (Fig. 7). Integrin beta4 (*ITGB4*) tends to skip exon 35 and *CD44 *[4,5] shows alternative splicing in about half the patient samples.

The relevant exons in *CD44 *did not score well in our detection analysis, probably because of the inconsistency in the splicing pattern. Densitometric analysis of *CD44 *(Additional file 5) confirms strong splice variation between the tissues, but with a high variability across samples. Note that the advanced tumor sample in Lane 13, the most predictive for our differential splice patterns, does not display alternative splicing of *CD44*.

*VEGF *has a potentially complex splicing pattern, with possible combinations of Ex6a, Ex6b, extended Ex6a and Ex7. McCullough et al. [30] attempted to resolve this complexity with a mass spectrometry-based method that indicated that the majority of the transcripts in normal colon lack Exon 6, whereas we find that about half of the transcripts migrate consistently with inclusion of Exon 6. The results from *CD44 *and *VEGF *underscore the difficulty of detecting relatively subtle alterations in small, heterogeneous sample populations.

### Pathway analysis of validated splicing events

**Figure 8:**
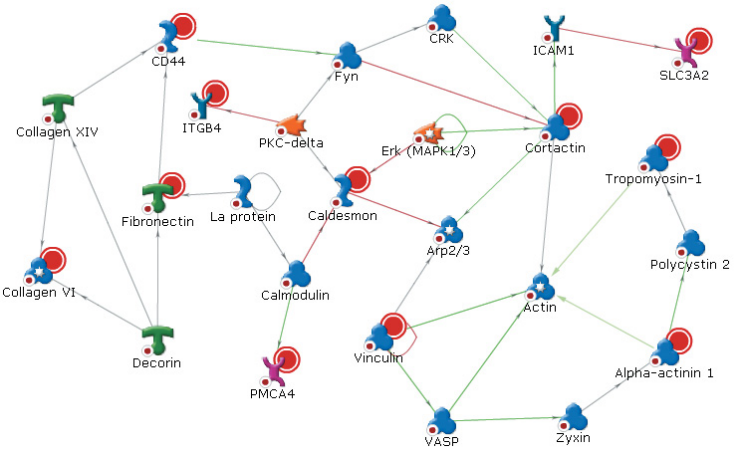
**Network containing products of alternatively spliced genes in colon cancer**. Proteins affected by alternative splicing are indicated with a red disk. Proteins concerned with cell or matrix adhesion are generally to the left while those concerned with cell motility and the actin cytoskeleton occupy the center and right. "PMCA4" is an alias for ATP2B4.

Analysis of the alternatively spliced genes with MetaCore software produced a network (Fig. 8) that can be roughly divided into two areas of cellular function. Cell motility and organization of the actin cytoskeleton functions include the alternatively spliced products of *TPM1*, *ACTN1*, *VCL*, *CALD1 *and *CTTN*, while cell adhesion and matrix organization includes those of *COL6A3*, *FN1*, *VCL*, *ITGB4 *and *CD44*.

### Comparison to bioinformatic methods of estimating alternative splicing

Several bioinformatic approaches have been developed to identify splice variation between normal tissue types or normal versus tumor tissues. These methods rely on mapping ESTs or mRNAs onto RefSeq transcripts or genomic sequence, making frequency counts of various isoforms, and applying a statistical test to detect differential inclusion of exons in different tissues. The methods vary somewhat in research focus, basic algorithms, statistical methods (e.g., Z-statistics vs. Fischer's exact test), EST source libraries, and mechanisms of normalizing or filtering out differential gene expression, but the essential approach is similar. We compared our empirical data from the exon array to three lists of the cancer-associated splicing events computationally predicted in Wang, et al. [31], the virtual SAGE-filtered list of Kirschbaum-Slager et al. [32] and in the BASD database described in Hui, et al. [33]. When evaluating the results, it must be kept in mind that these bioinformatic approaches assay either tumors generally or a range of tumor types, but not specifically colon cancer (as in our study).

The splicing events derived from the three methods were mapped to Unigene gene symbols where possible and compared to the top 200 candidate splicing events from the exon array, which corresponds to 169 genes (see Additional file 3). Our list of candidates matched approximately an equal proportion of each EST-derived list, between 1.92% and 1.97% of the total genes in each set: 12/625 for Kirschbaum-Slager et al., 33/1733 for BASD and 13/660 for Wang et al. Altogether, 53 of our 169 candidate genes are found in at least one list. A simple chi-squared test indicates that elements of each list are highly overrepresented in each of the other lists (with p-values < 0.01). The strongest association between the exon-array candidates was with the BASD list, most likely because of the increased statistical power of this larger data set. The EST-based prediction sets showed a similar distribution of association to each other as our candidate gene list did to each of them.

Among our 14 validated candidates, three (*CALD1*, *FN1*, *TPM1*) appeared in the BASD list, two (*CALD1*, *GK*) appeared on the list of Wang et al., and none appeared on the list of Kirschbaum-Slager et al. Of the six previously reported alternatively spliced genes tested here, two (*CD44 *and *VEGF*) are found in BASD, two (*MST1R *and *VEGF*) are found in the list of Wang et al. and one (*RAC1*) appears on the list of Kirschbaum-Slager et al. *CD44*, *SIAHBP1 *and *VEGF *do appear in our list of 169 candidate genes, but none ranked in the top 100.

## Discussion and Conclusion

There are many design differences between the exon array and the U133 Plus 2: feature size, the number of probes per probeset, the number of probesets per transcript, background calculation, the assay procedure, etc. The most obvious difference is that the exon array interrogates exons/subexons throughout the transcript, while the U133 Plus 2 generally targets only the 3' end. Alternative splicing and, particularly, alternative polyadenylation sites may account for substantial differences in gene signal estimation. Furthermore, our method of averaging the signal value of multiple probesets targeting the same transcript on the U133 Plus 2 is only a rough compromise. Despite the many differences, the gene-level comparison demonstrated a reasonable correlation in signals for genes that are significantly different between tissue types. The most notable difference in signal estimations is the shift of apparently low expressing genes on the U133 Plus 2 array to higher signals on the exon array. Since the exon array generally contains 3 to 4 times as many probes per transcript as the U133 Plus 2 array and the probesets are distributed throughout the transcript cluster, the exon array may be more sensitive in detecting a subset of low expressing genes, at least in this data set. Resolution of, for example, the portability of results between the arrays types, or the characteristics of transcripts that are differentially detected, will require several dedicated studies, for which data is becoming available.

Among the 160 genes differentially expressed in the Core gene set, 60 have been previously identified as participating in cancers, with 21 specifically in colon or colorectal cancer. Almost one third of the up-regulated genes are part of a tightly interconnected network involved in mitosis, cell cycle control, cell proliferation, invasion, matrix remodeling and Wnt signaling. Constitutive activation within the Wnt signaling pathway has been a prevalent theme in colon cancers, in particular the role of β-catenin [18,34]. Eight of the over-expressed genes here (*BIRC5*, *MMP7*, *VEGF*, *ENC1*, *CCND1*, *STRA6*, *MET *and *CLDN1*) are targets of Wnt/β-catenin regulation and two other Wnt-associated genes, *SOX9 *and *HIG2*, are up-regulated in these colon cancer samples.

Twenty nine of the tumor-expressed genes have unknown or weakly annotated functions, but several may be involved in cell proliferation or apoptosis (*MCTS1*, *SIL*, *HSPA5BP1*, *WDR18 *and *IFITM1*) and at least eight have previously been associated with various cancers (Additional file 1). Thirteen putative transcriptional regulators and most of the genes classified as signal transducers have unknown biological roles. Another seven genes annotated only as "hypothetical protein" or "open reading frame" (*MGC4677*, *C12orf11*, *FLJ10726*, *C6orf167*, *FLJ31153*, *FLJ20272*, *DKFZp762E1312*, *KIAA1217 *and *FLJ10719*) are strongly expressed above background. The expanded analysis based primarily on *ab-initio *exon predictions identified 38 more genes with significant expression even though they completely lack previous annotation (Additional file 2). These transcripts with unknown functions may represent a novel set of targets for study in colon cancer oncology and demonstrate the exploratory power of an inclusive array design.

We have identified a number of genes that are differentially spliced between normal and cancerous tissue. Most of the tissue-specific alternative splicing events that were experimentally validated occurred in genes involved in cytoskeletal structure, the extracellular matrix or cell-cell interactions. Some of these events are reported splice variants that occur in a tissue-specific manner and may represent a loss of tissue function as colonic epithelial and smooth muscle cells dedifferentiate, rather than abetting transformation or metastasis. Determination of the role of these splice events requires more detailed study, but in most cases these genes have previously been implicated with active roles in the progression of tumors.

Five of the genes (*TPM1*, *ACTN1*, *VCL*, *CTTN *and *CALD1*) that we found to be alternatively spliced in colon cancer have actin-binding domains and play a direct role in the organization or structure of the cytoskeleton. Remodeling of the cytoskeleton is fundamental in proliferation, apoptosis, cell invasion and metastasis [35].

TPM1 appears to act as a tumor suppressor by promoting anoikis (apoptosis induced by cell detachment) [36]. Down-regulation of *TPM1 *by oncogenic transformation results in a loss of actin stress fibers [37,38], whereas restoration of *TPM1 *inhibits cell migration in metastatic cell lines [39]. A splice variant of one of the low molecular weight isoforms of tropomyosin has been found specifically in association with colonic polyps and adenomas, but not normal colon tissue [40].

Actinin is a component of stress fibers and links the cytoskeleton to adherens-type junctions. It plays a role in cell migration probably by facilitating detachment of focal adhesions distal to the direction of movement [41]. Alternative splicing of actinin-4, which has a high sequence similarity to ACTN1, apparently leads to an abnormal cytoskeleton in small cell lung cancer [42]. ACTN1 also has a binding site for VCL, and the two proteins cooperate to organize the cytoskeleton at adhesion junctions [43].

CALD1 binds actin and responds to calmodulin to promote stress fibers and focal adhesions, and CALD1-defective cells are highly impaired in motility [44]. In cells transformed by Kaposi sarcoma-associated herpes virus (KSHV) or v-*erb*B, hypermethylation of *CALD1 *and recruitment of its product into membrane complexes is linked to the loss of cytoskeletal microfilaments [45,46].

CTTN, frequently overexpressed in breast cancer and squamous cell carcinomas, is highly enriched at tumor invasion fronts [47,48]. Two conformational forms of CTTN are known, with both forms present in normal cells, but the apparently larger one (p85) is more prevalent in colorectal cancers [47]. Two splice variants that affect cell mobility have been previously identified: SV-1 (lacking Exon 11) and SV-2 (lacking Exons 10 and 11). SV-1 and full-length *CTTN *were equally abundant in normal cells while SV-2 was barely detectable. SV-1, but not SV-2, can bind and crosslink actin, but overexpression of either form interferes with cell migration [49]. Our results indicate that transcripts with or without Exon 11 are approximately equal (in agreement with previous data), but transcripts carrying Exon 11 (i.e., full length) are relatively more abundant in colon cancer samples, suggesting that these cells may be more competent for motility.

Alternative splicing of *FN1*, *CD44 *and *COL6A3 *may play some role in matrix remodeling and/or cell migration in colon cancer, though *COL6A3 *has not been previously implicated in this role. Fibronectin was one of the consistently up-regulated genes in an artificial selection for highly metastatic cell lines, which also identified *ACTN1 *and several collagens [50]. Two splice variants of *FN1 *have been implicated in the neo-vasculature of a variety of human tumors but not in normal adult tissues, however the role of these species in tumor-related angiogenesis is unclear [51]. Alternatively spliced *FN1 *containing an extra domain has been found frequently in cancers [3], whereas we find preferential skipping of Exon 25 in tumor tissues. CD44 is involved in both cell-matrix and cell-cell interactions as well as connections to the actin cytoskeleton via ankyrin. The variably spliced region of *CD44 *(exons 6–15) is preferentially included in many cancer types and appears to affect cell migration, invasion and metastasis [5].

Integrin ITGB4 interacts with the intermediate filament network, stimulates the Ras and PI3-K signaling pathways, and appears to be important for cell invasion in colon cancer [52,53]. SLC3A2, which functions in transmembrane transport, associates with integrins and appears to participate in integrin-mediated anchorage-independent cell growth and tumorigenesis in 3T3 fibroblasts [23,54]. The inclusion of several exons of *SLC3A2 *in colon cancer transcripts may represent a tumor-specific alteration of its role in integrin signaling.

Of the eleven differentially spliced genes we found or confirmed, ten are involved in the organization of the cytoskeleton or interaction with the matrix or other cells. Seven of these genes (*TPM1*, *CALD1*, *CTTN*, *FN1*, *CD44*, *ITGB4 *and *SLC3A2*) have previously been implicated in cancers, and, in five cases, specific splice variants are correlated with the cancerous state. This grouping may represent a coherent, and possibly coordinated, set of alterations which may impact cell mobility and extracellular interactions. A similar concentration of splice variants was found in mouse brain, where targets of the Nova splicing regulon are clustered into functions affecting synaptic transmission and cell morphology. In fact, the targets appear to act as a modular network that impacts not only signaling functions, but also specifically the actin cytoskeleton, extracellular matrix and cell-cell adhesion [55]. It is possible that a similar splicing network is altered in colon cancer to produce the complex of interacting splice variants seen in this study. Such patterns may be more apparent in a highly parallel genome-wide exon analysis than in traditional methods involving gene-by-gene searches.

One assumption of this type of analysis is that biologically important splicing changes would consistently appear in samples of a particular category (i.e., colon cancer). In some cases, our samples did not reproduce some previously reported events (*Rac1*, *VEGF*, *SIAHBP1*, and *MST1R*), or else the changes were sporadic (*CD44*). Furthermore, we find differences even in normal samples with regard to Exon 6 of *VEGF *compared to the mass spectrometry results of McCullough et al. [30]. In fact, the most relevant splicing change in *VEGF *for tumorigenesis may be a splice variant involving Exon 8 [56]. In this situation, it may be difficult to resolve what the normal and abnormal states are for *VEGF*. Inconsistent results may be due to differences in samples, experimental procedures, analyses or interpretations, suggesting that conclusions about subtle changes in splicing may need to be reinforced by multiple sources. On the other hand, many of our validated splicing events have been observed in several other instances. Nearly identical patterns of differential alternative splicing was found in colon tumors by Okumura et al. for *TPM1*, *ACTN1 *and *ITGB4*, and these patterns were even more emphatic in tumor cell lines. Interestingly, differential splicing of *CD44 *in colon cancer was not observed in that study [57]. *ITGB4 *and *TPM1 *were identified by an EST-based analysis, and experimentally validated as differentially spliced in several tumor types [58]. The alternative splicing of *CTTN *is consistent with protein alterations seen in association with colon cancer [47]. Our sample set ranged from well differentiated to poorly differentiated (i.e., advanced) tumors, yet *ACTN1*, *ATP2B4*, *VCL *and *CALD1 *show strong and consistent changes across our samples (Additional file 5). The presence of these splice variants in multiple tumor stages argues that they must be both early and persistent events. Finally, differences in splicing patterns may be due to biologically important differences in cancer etiology, and therefore be useful indicators of tumor subtypes or stages.

With the large amount of EST and genomic data available, a great deal of useful information may be gained from *in silico *prediction of transcript isoforms. Validation rates from such methods have been fairly high and the resulting pattern of predictions are mostly in line with empirical data. There are a number of potential hazards in this approach: EST libraries are highly variable in quality and reliability, it is difficult to account for differential gene expression, and tissues and individual genes are unevenly represented. Almost 45% of EST libraries are from cancer samples [33] and 70% of mRNAs in GenBank are cloned from tumor samples [59], leading to a strong bias against identifying isoforms present in normal tissues. Also, in spite of various methods to remove or normalize differentially expressed genes, this factor may lead to overprediction [32]. The exon array addresses many of the difficulties by providing an empirical platform that is unbiased with regard to tissue or gene representation, and allows for direct normalization of differential gene expression. While a substantial proportion (32%) of our top candidates for alternative splicing matched predictions from three EST-derived methods, only four of our validated splicing events appeared among the 2797 genes with bioinformatically predicted splice variants. This suggests that there are substantial gaps in current EST databases that must be addressed empirically.

The splicing changes seen here are not necessarily as dramatic as seen in a previous study of 16 pure normal tissues with an exon-based microarray (Clark *et al*., in prep.). This highlights the subtlety of most splicing events, which are typically not an all-or-none phenomenon, so sample size and homogeneity are important considerations. In spite of limitations which are frequently encountered in cancer and other tissue studies in humans, such as a modest sample size, heterogeneous tissues, and multiple categorical variables (tumor stage, gender, and individual patient variation), we were able to identify and validate a number of candidate colon cancer-specific splicing events. Exploration of alternative splicing will promote understanding of cancer etiology and may provide therapeutic targets and diagnostic markers [2,3].

## Methods

### Sample preparation and array hybridization

Ten matched-pairs (20 total RNA samples) of human colon primary tumor and adjacent normal tissue Total RNA (P/N R8235090-PP-10) were purchased from Biochain Institute, Inc. (Hayward, CA). Tumors were characterized as poorly (2 samples), moderately (4) or well (4) differentiated. Adjacent normal tissue may include both colonic epithelial and smooth muscle tissue. Patient information and associated .CEL files are available from Affymetrix, Inc. [60].

One μg of each sample was processed using a pre-commercial version of the Affymetrix GeneChip Whole Transcript Sense Target Labeling Assay [61]. Pre-commercial versions of the GeneChip WT cDNA Synthesis Kit, WT cDNA Amplification Kit, and WT Terminal Labeling Kit (Affymetrix, Inc., Santa Clara, CA), containing identical formulations to the commercial kits, were used for target preparation. Eight μg of cRNA were input into the second-cycle cDNA reaction. Hybridization cocktails containing 3 to 4 μg of fragmented, end-labeled cDNA were prepared and applied to GeneChip Human Exon 1.0 ST arrays. Hybridization was performed for 16 hours using the MES_EukGE-WS2v5_450-DEV fluidics wash and stain script (pre-commercial FS450_0001 script). Arrays were scanned using the Affymetrix GCS 3000 7G and GeneChip Operating Software v. 1.3 to produce .CEL intensity files.

### Data analysis algorithms

Signal estimates were derived from the CEL files of the 20 samples by quantile sketch normalization using the PLIER algorithm for probeset (exon-level) intensities and IterPLIER for gene-level intensities [11]. Presence/absence of exons was determined by "Detection Above Background" (DABG), using surrogate GC mismatch intensities.

Candidate exons for alternative splicing were detected using a Splicing Index and the MIDAS algorithm [11]. The Splicing Index (SI) represents the log ratio of the exon intensities between the two tissues after normalization to the gene intensities in each sample: SI_i _= log2((e_1i_/g_1j_)/(e_2i_/g_2j_)), for the i-th exon of the j-th gene in tissue type 1 or 2. The splicing indices are then subjected to a t-test to probe for differential inclusion of the exon into the gene. MIDAS employs the Splicing Index in an ANOVA model to test the hypothesis that no alternative splicing occurs for a particular exon. The ExACT program implements the MIDAS algorithm and is available for download. Default parameters were used in all algorithms.

### Data analysis

ANOVA p-values and fold changes for gene expression were calculated using Partek Genomic Suite 6.2 (Partek Inc., St. Louis, MO) and biochemical/regulatory network mapping of significant genes was performed using MetaCore 3.2.1 software (GeneGo Inc., St. Joseph, MI). Patient 3 was removed as an outlier due to aberrant behavior in PCA, which improved the signal-to-noise ratio for discrimination by tissue type. Noise ratios also showed that gender, patient and tumor differentiation categories were relevant as factors in the analysis.

For efficient functioning of the alternative splicing algorithms, exons and genes with low expression were removed from the analysis by the following criteria (see Fig. 4). Exons were accepted if they had a DABG p-value < 0.05 in at least 50% of the samples in either tissue. Genes were accepted if either: a) at least 50% of the core exons in at least 50% of samples in both tissues had a DABG p-value < 0.05 or b) if the IterPLIER gene-level signal intensity exceeded a particular threshold, depending on the filtering version. The gene-level requirement is necessary because alternative splicing is meaningless if the gene is not significantly expressed in both tissues.

Subsequent to filtering, the gene-level and exon-level PLIER intensities were jointly processed by either MIDAS or SI. Exons from the Full meta-probeset that associate with these Core genes were included in the analysis. The results were filtered for a Slicing Index > 0.5, and high scoring hits were taken for further analysis. Candidate exons were visually inspected in genomic context (Integrated Genome Browser, Affymetrix Inc. or BLIS 6.2.1, Biotique Systems Inc., Reno, NV) to ensure that exon-level expression patterns were consistent with potential alternative splicing. For example, exon intensities that strongly and uniformly deviate from that of adjacent well-annotated exons in the same gene may indicate cross hybridization, poorly performing probes or absent target. Visualization at the probeset level is also useful to insure that the signals are consistent within tissue groups, that neighboring constitutive exons are comparably expressed and that multiple probesets interrogating the same exon have similar signals.

Finally, possible splicing patterns were determined by mapping the probesets to publicly available mRNA and EST sequences on the UCSC Genome Browser [62] via BLAT. Candidates consistent with known examples of alternative splicing are more likely to prove to be true positives, but this approach will also suppress the discovery of novel splicing events. In addition, the BLAT results indicate whether there similar sequences elsewhere in the genome that may lead to false signals by crosshybridization.

### Array Comparison

For comparison of gene-level expression between the Exon Array and the Human Genome U133 Plus 2.0 Array, total RNA from 11 different tissues were purchased from Ambion, Inc. (Austin, Texas). In brief, three assay replicates were prepared from each tissue with 5 μg (3' IVT) or 1 μg (WTA) of RNA according to the Expression Analysis Technical Manual or the Whole Transcript Sense Target Labeling Assay Manual, and hybridized to the Exon and the U133 Plus 2 arrays. Signal estimations were generated with PLIER, using the Core meta-probeset in the case of the Exon Array. The signals were quantile normalized within tissues and then median normalized across the tissue types. The signal was transformed and variance stabilized by log_2_(signal +16). The CEL files and signal estimation data are available from Affymetrix [60]. A 1-way ANOVA was performed with the Partek Genomics Suite across all tissue types. P-value cutoffs were determined by Bonferroni multiple test correction with thresholds at 0.05, 0.001 and 10^-8^. Genes which were significantly different in any pair of tissue types were kept for further analysis. Transcript clusters (Exon) and probesets (U133 Plus 2) were mapped to each other according to the file "HuEx-1_0-st-v2 Transcript Cluster ID Mappings to HG-U133 Plus 2.0" [63]. Multiple U133 Plus 2 probesets which target the same transcript cluster were averaged to obtain the signal estimate. The subsequent comparisons were generated with the Partek Genomics Suite.

### RT-PCR validation

Approximately 2 μg of total RNA was reverse transcribed to cDNA using the TaqMan Reverse Transcription Reagents Kit per manufacturer's instructions (Applied Biosciences) with a combination of oligo dT and random hexamers as primers. PCR was carried out for 35 cycles using Taq Polymerase (Promega) per manufacturer's instructions with approximately 50–100 ng of cDNA as template. PCR products were separated on 2.5% agarose gels and stained with ethidium bromide for visualization. All RT-PCR primer sequences are included in Additional file 4.

In order to discriminate between the two mutually exclusive exons (Ex7 & Ex8) of the same size in the TPM1 gene, the resulting PCR products were distinguished with an enzyme that cuts only one of the exons. 30 μl of PCR product was digested with 1 U of PvuII (New England Biolabs) at 37°C for 1 hour per manufacturer's instructions. PvuII cuts a sequence (CAGCTG) present in Ex7, but not in Ex8. Thus, RT-PCR products generated from mRNAs containing Ex7 are cut into two smaller, faster migrating segments when separated on a 2.5% agarose gel.

Densitometric analysis of the RT-PCR gels was carried out using AlphaEaseFC 4.0 (Alpha Innotech Corp., San Leandro, CA) software per manufacturer's recommendations. A ratio of two isoforms was generated using the sum of all pixel intensities within an identically sized region containing each band. In some cases, where appropriate, intensities from multiple bands were added together to generate a single value representing an isoform. In order to determine if the change in isoform expression was statistically significant, a simple two-tailed t-test was carried out on the isoform ratios by grouping the 10 samples from either "tumor" or "normal" tissue. A summary of the results is included in Additional file 5.

## Authors' contributions

BS processed samples and performed hybridizations on GeneChip^®^. TC, MS, QY and SD performed the PCR validation. PG, TC, AS, YT, JV, CS, CD and AW performed data analysis and algorithm development. TA participated in experimental design and interpretation. The manuscript was drafted by PG and YT. All authors read and approved the final manuscript.

## Supplementary Material

Additional file 1 **Table_A1_differential_CORE_genes**. Genes in the Core metaprobe set which are differentially regulated in colon cancer. The log fold change is shown for Normal relative to Tumor samples, which is negative for up-regulated genes. "Cancer" Indicates that the gene has been implicated in various cancers. Question marks indicate an unproven association. "CRC" indicates that the gene has been implicated specifically in colon or colorectal cancer. Gene annotations are according to the Affymetrix file HuEx-1_0-st-transcript-annot.csv (NCBI build 35, UCSC hg17). Abbreviations: Exc., extracellular; proc., processing; R & R, recombination and repair. Click here for file

Additional file 2 **Table_A2_differential_FULL_genes**. Genes in the Full metaprobe set which are differentially regulated in colon cancer. Transcript IDs and gene annotations are according to the Affymetrix file HuEx-1_0-st-transcript-annot.csv (NCBI build 35, UCSC hg17). Genes which are also present in the Core list are omitted. "Support" indicates genes that have prior either annotation ("A") or a transcript expression exceeding a threshold PLIER signal of 15 in 50% of the samples in at least one tissue type ("E"). Click here for file

Additional file 3 **Table_A3_AS_200Candidates**. Top scoring candidates for alternative splicing events. This list was obtained by a) filtering exons with DABG at 0.05, b) filtering genes at a signal threshold of 70, c) requiring an log ratio > 0.5, d) a Splicing Index p-value < 0.005 and e) only Core exons (Note that the list extends to the top 200 events that were used for comparison to bioinformatic EST methods). The Exon ID and Gene ID are the Affymetrix probeset IDs and transcript cluster IDs, respectively. Log mean difference is the logged ratio of the exon signal from each tissue after normalization to gene-level signals in each tissue. SI p-value is derived from the Splicing Index t-test. Click here for file

Additional file 4 **Table_A4_PCR_primers**. Primers and summary of PCR results for splicing candidates and previously reported splicing events in colon cancer. Forward and Reverse are the exon locations of the validation PCR primers. "Known Alternative Splicing Events" were determined from RefSeq, full length mRNAs and ESTs on the UCSC Genome Browser. "Result" indicates the interpretation of the PCR result ("NC", No change in alternative splicing between the two tissue types; "No", no alternative splicing observed; "GE", differences attributed to different levels of gene expression in the two tissue types). Abbreviations: Ex, exon; CE, cassette exon; MEE, mutually exclusive exon; Alt., alternative. Click here for file

Additional file 5 **Table_A5_PCR_densitometry**. Analysis of densitometric scan data from PCR validation gels. Click here for file

## Figures and Tables

**Table 1:** Differentially spliced genes in colon cancer detected by the exon microarray and confirmed by PCR.

**Gene**	**F**^1^	**R**^1^	**Known AS Event**^2^	**Confidence**^3^	**Preferential Inclusion of Splice Variants**^4^	**FC**^5^
ACTN1	Ex18	Ex20	19a/19b are MEEs	Good	Ex19a in Tumor, 19b in Normal	-1.05
ATP2B4	Ex20	Ex22	Ex21 is CE	Good	Ex21 in Normal	+1.53
VCL	Ex18	Ex20	Ex19 is CE	Good	Ex19 in Normal	+1.22
CALD1	Ex5	Ex7	extended Ex5; Ex6 is CE	Good	extended Ex5 + Ex6 in Normal; Ex6 in Normal	+1.36
SLC3A2	Ex1	Ex5	Ex2, 3, 4 are CEs	Good	Normal skips all exons	-1.90
COL6A3	Ex2	Ex5	Ex3 and Ex4 are CEs	Good	Ex3 or Ex4 in Tumor	-1.56
COL6A3	Ex5	Ex7	Ex6 is CE	Good	Ex6 in Tumor	-1.56
CTTN	Ex10	Ex14	Ex11 is CE	Good	Ex11 in Tumor	-1.21
FN1	Ex24	Ex26	Ex25 is CE	Good	Ex 25 in Normal	-1.86
TPM1	Ex6 Ex6	Ex7 Ex8	Ex7/Ex8 are usually MEEs	Good	Ex7 in Tumor; Ex8 in Normal	+1.92
GK	Ex19	Ex21	Ex20 is CE	Mod.	Ex20 in Tumor	-1.01
MAST2	Ex7 Ex5	Ex9 Ex9	Ex7 alt. start?	Mod.	Ex7 Alt. Start in Normal	+1.26
LGR5	Ex10 Ex11b	Ex12 Ex12	extended Ex11; Ex11b is CE	Weak	extended Ex11 + Ex11b in Tumor	-12.1
ZAK	Ex11	Ex11b	Ex11 Alt. 3' end	Weak	Alt. 3' end (Ex11b) in Normal	-1.07
ZAK	Ex16	Ex18	Ex17 appears constitutive	Weak	different 3' end of gene	-1.07
FXYD6	Ex1	Ex4	Ex2 and Ex3 are rare CEs	Very Weak	Ex2 + Ex3 in Tumor	+2.50

^1 ^Exon locations of the F (forward) and R (reverse) PCR primers. ^2 ^Known Alternative Splicing Event As determined from RefSeq, full length mRNAs and ESTs on the UCSC Genome Browser. ^3 ^Relative confidence in the interpretation. ^4 ^Interpretation of the PCR results in terms of transcript structure. ^5 ^Fold change at the gene level. (-) means that the signal is lower in Tumor. Abbreviations: Ex, exon; CE, cassette exon; MEE, mutually exclusive exon; Alt., alternative.
